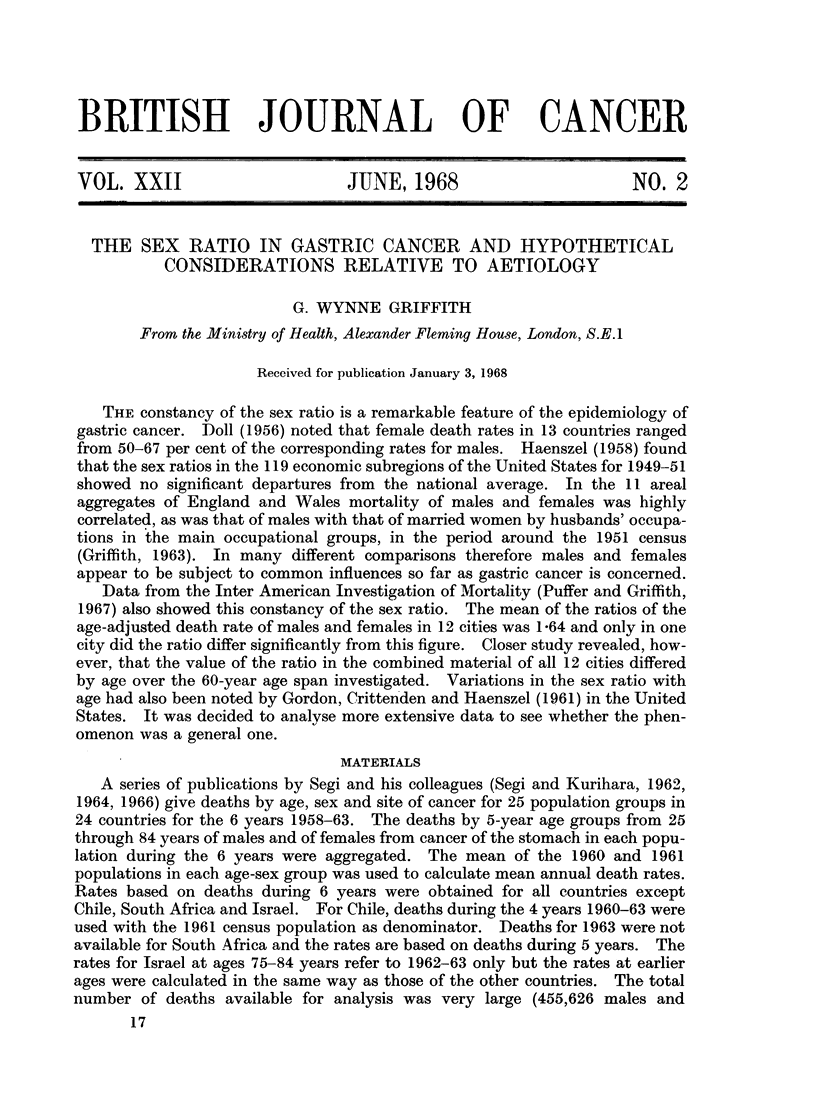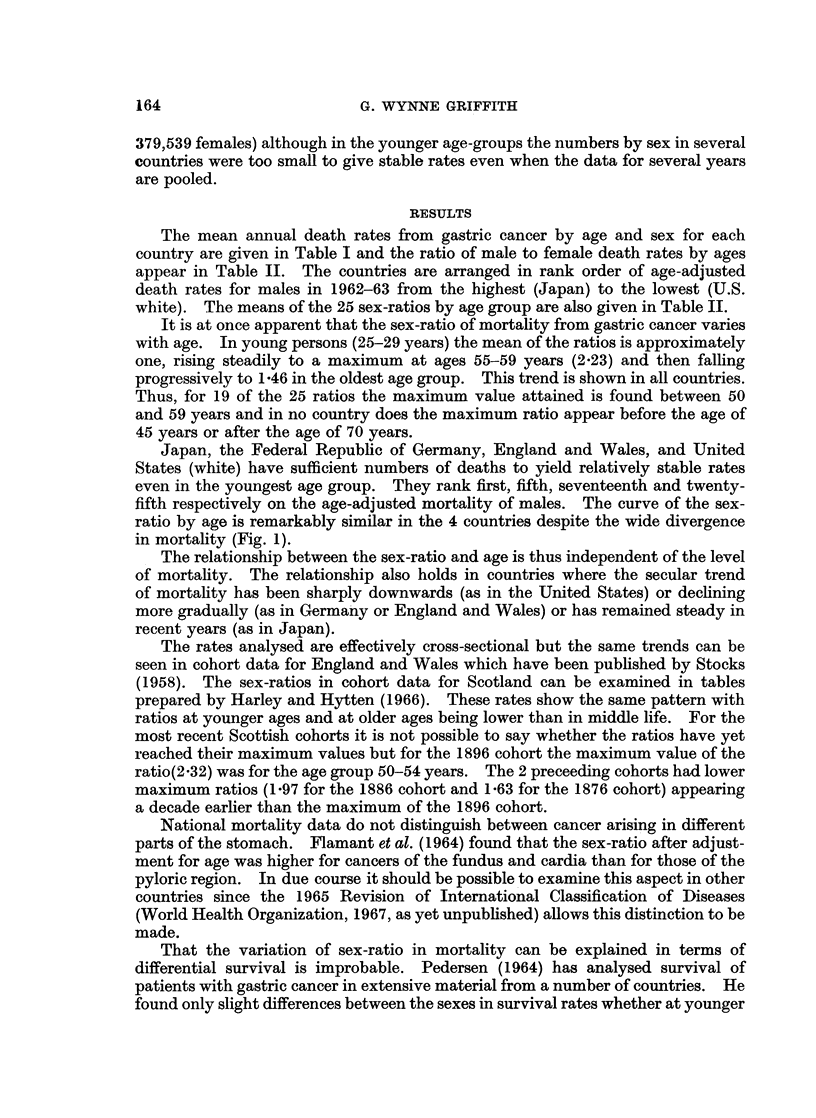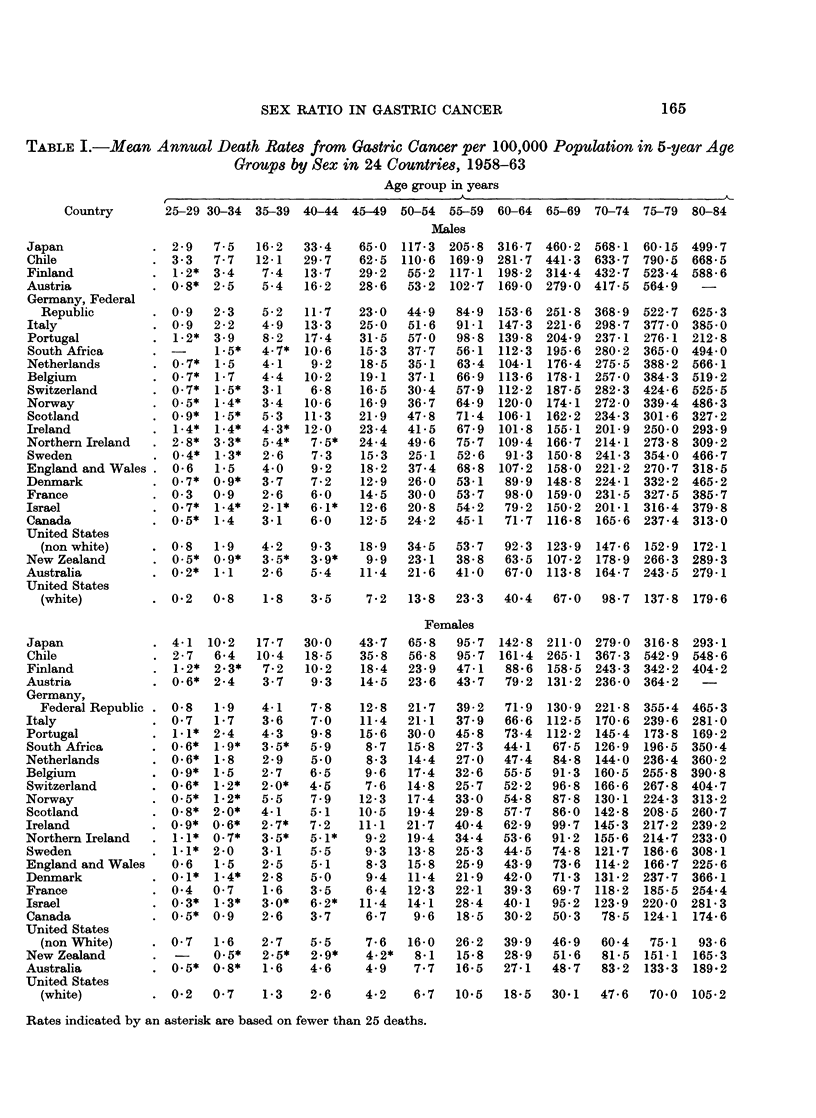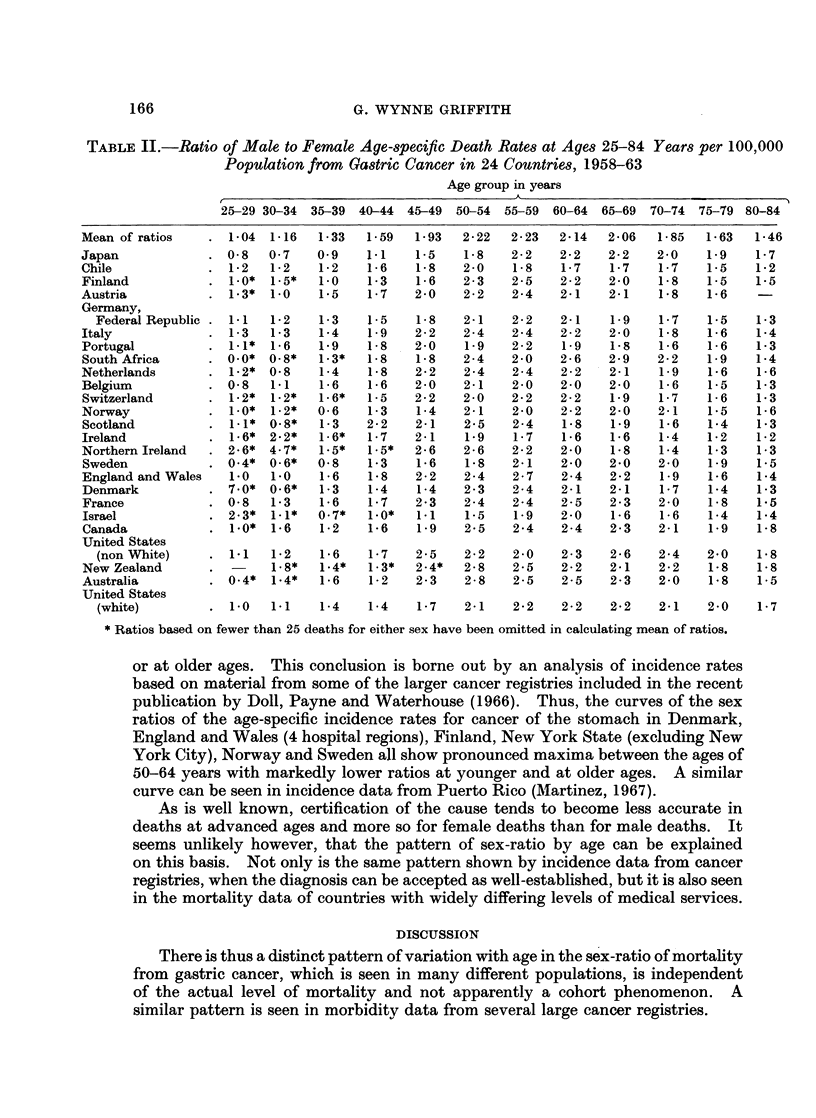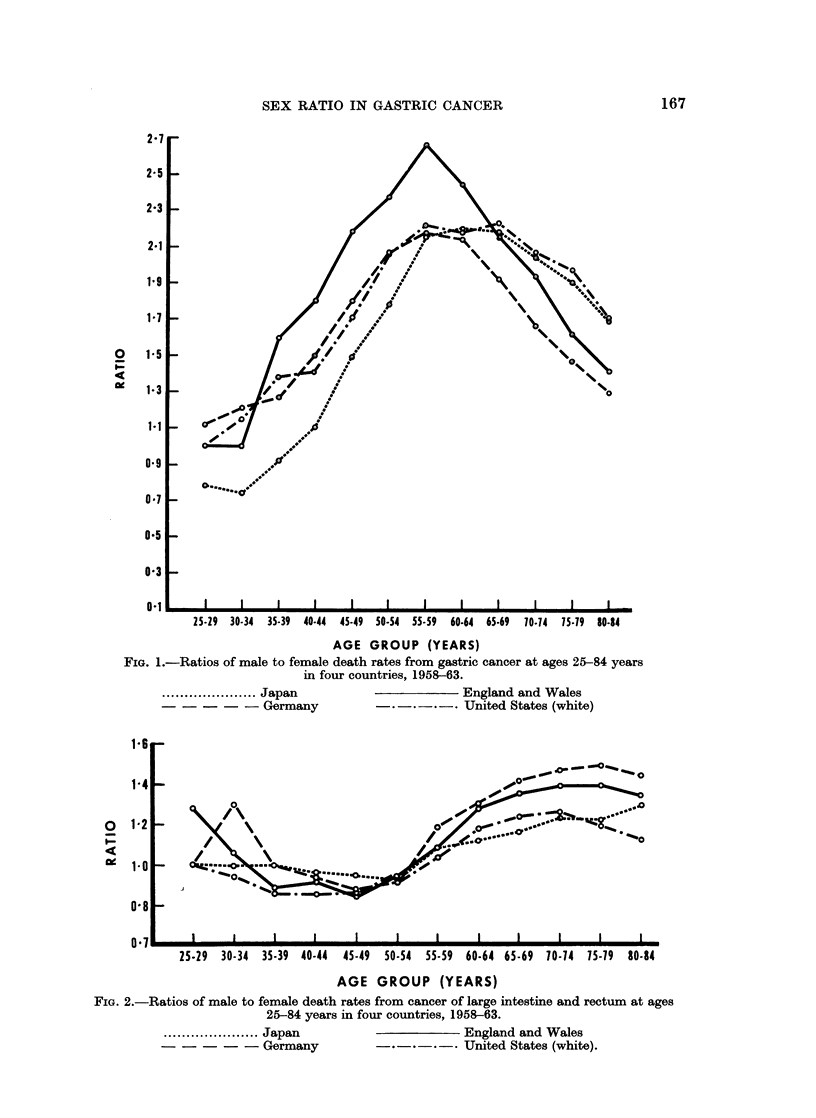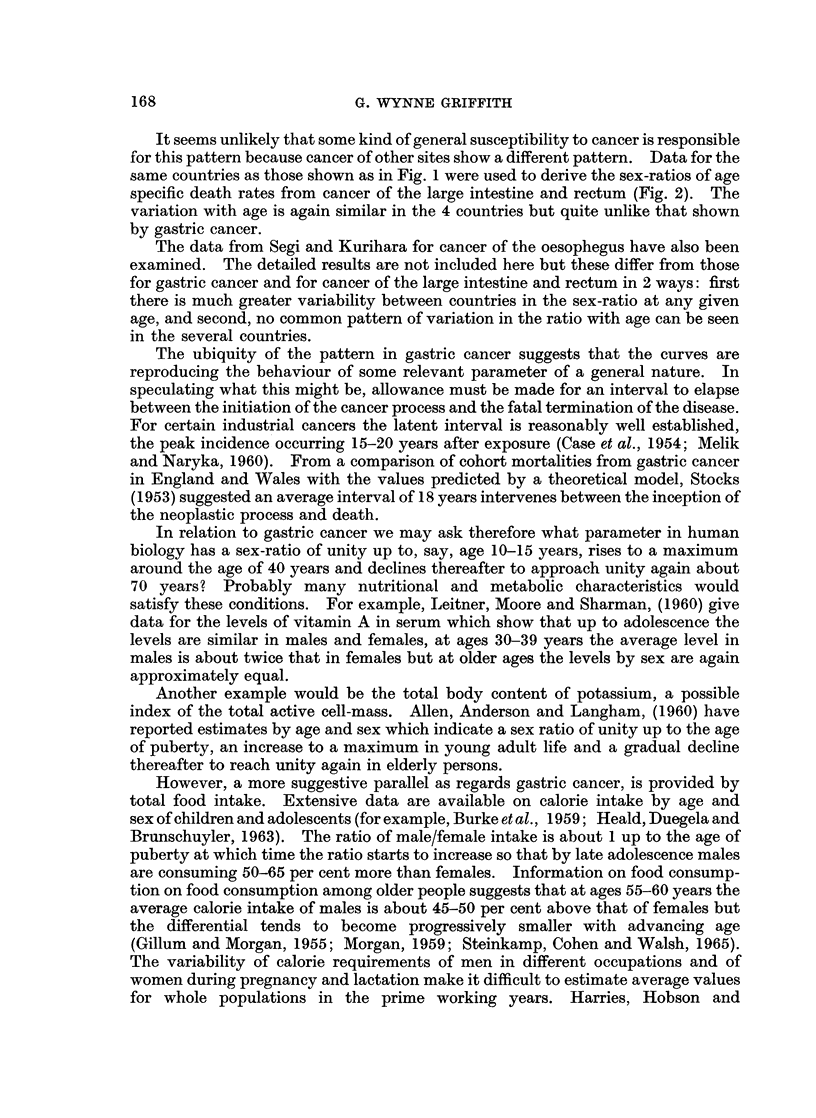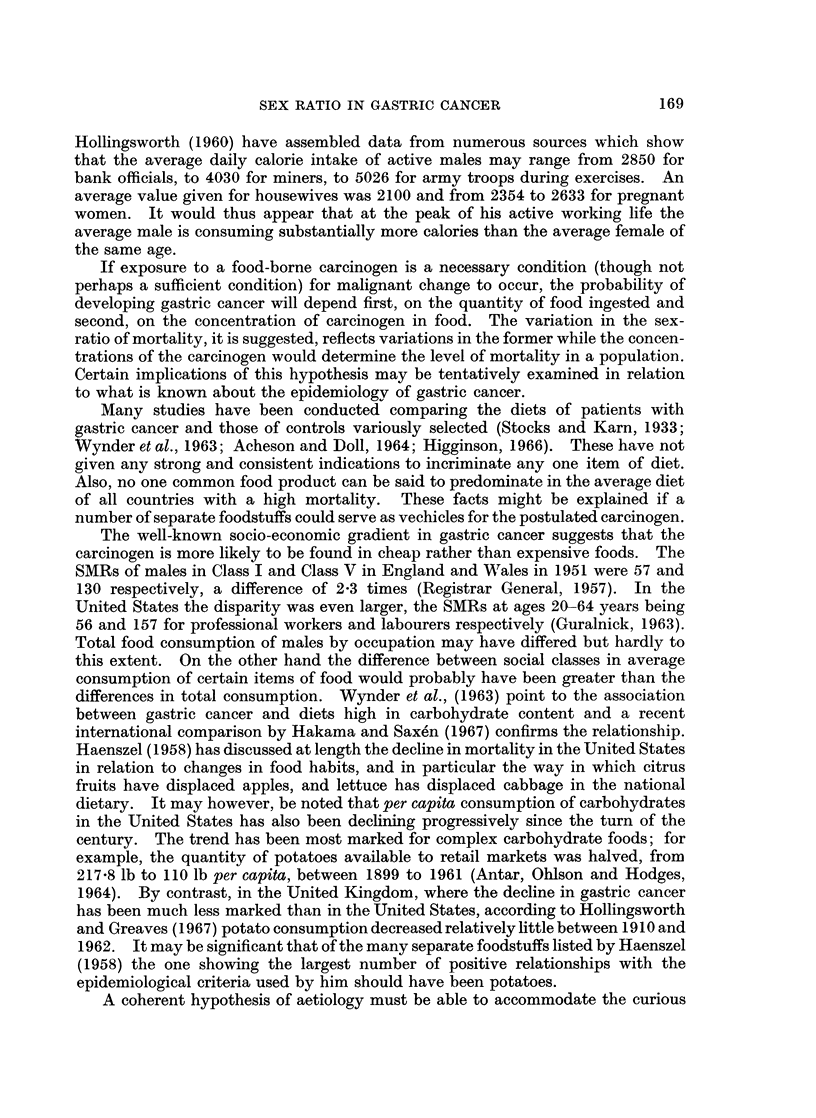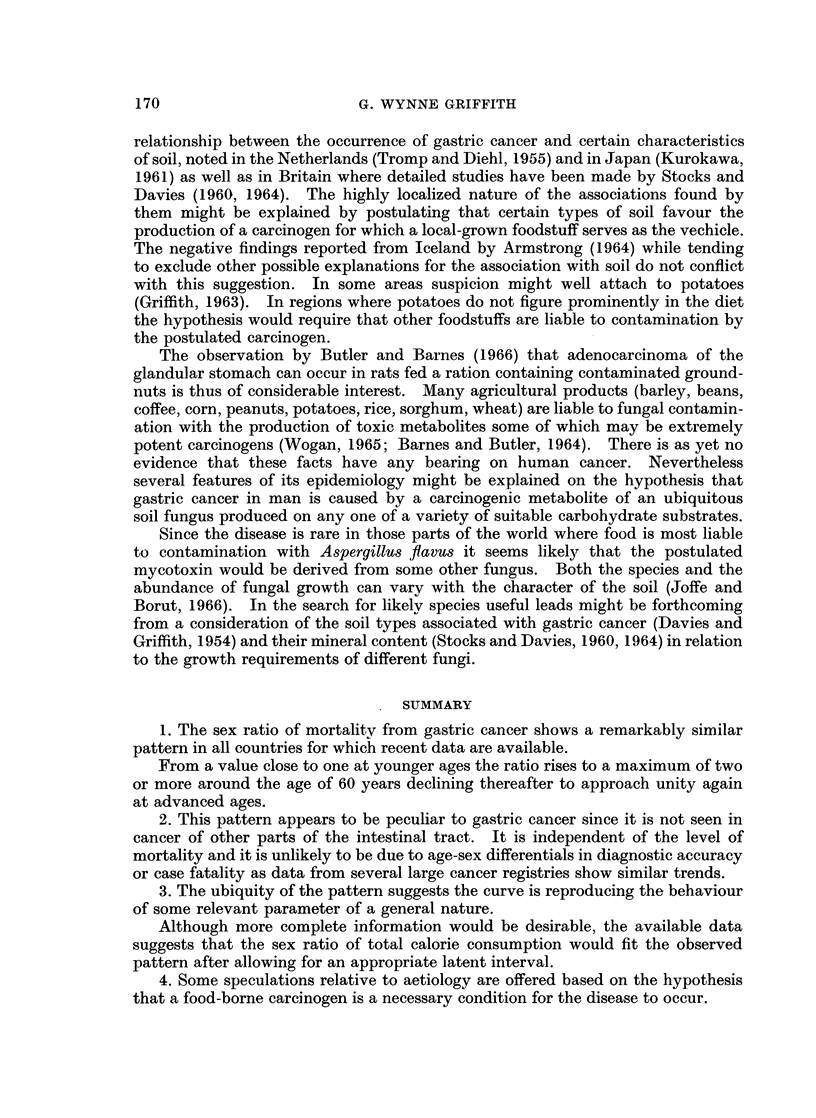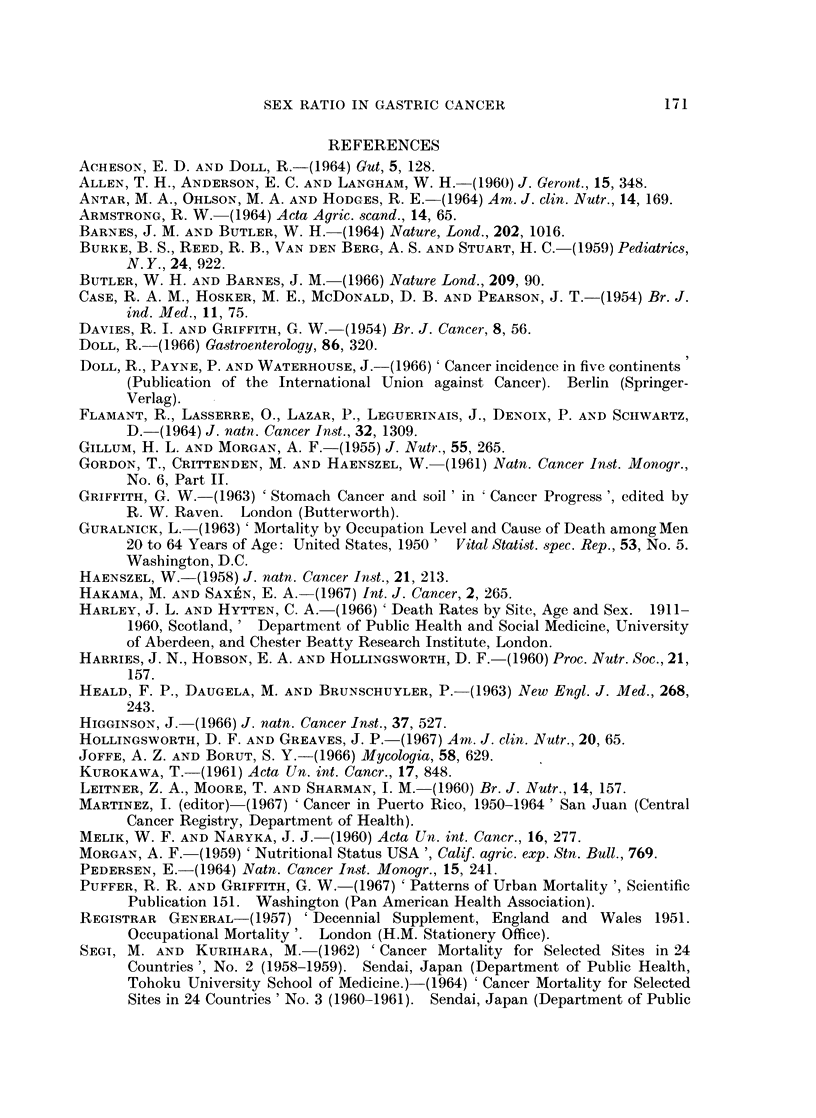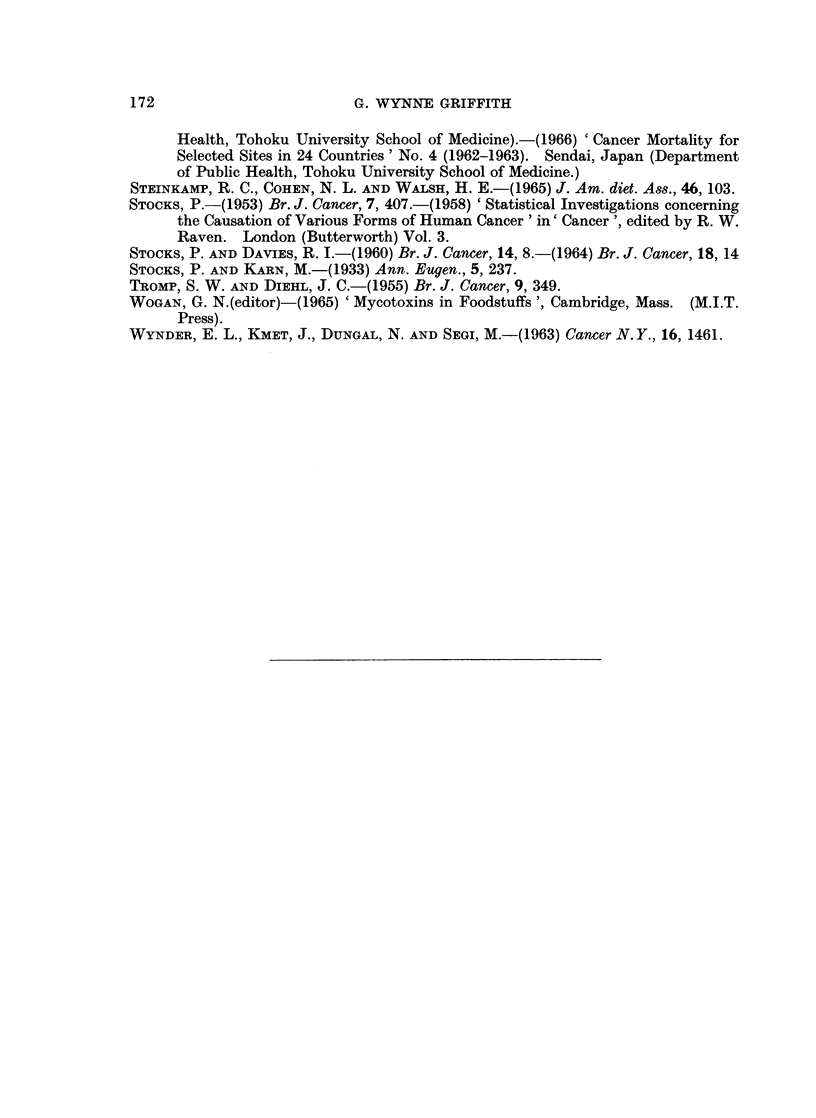# The sex ratio in gastric cancer and hypothetical considerations relative to aetiology.

**DOI:** 10.1038/bjc.1968.23

**Published:** 1968-06

**Authors:** G. W. Griffith


### BRITISH JOURNAL OF CANCER

VOL. XXII JUNE, 1968 NO. 2

### THE SEX RATIO IN GASTRIC CANCER AND HYPOTHETICAL CONSIDERATIONS RELATIVE TO AETIOLOGY

G. WYNNE GRIFFITH

*From the Ministry of Health, Alexander Fleming House, London, S.E.*1

Received for publication January 3, 1968

THE constancy of the sex ratio is a remarkable feature of the epidemiology of gastric cancer. Doll (1956) noted that female death rates in 13 countries ranged from 50–67 per cent of the corresponding rates for males. Haenszel (1958) found that the sex ratios in the 119 economic subregions of the United States for 1949–51 showed no significant departures from the national average. In the 11 areal aggregates of England and Wales mortality of males and females was highly correlated, as was that of males with that of married women by husbands' occupations in the main occupational groups, in the period around the 1951 census (Griffith, 1963). In many different comparisons therefore males and females appear to be subject to common influences so far as gastric cancer is concerned.

Data from the Inter American Investigation of Mortality (Puffer and Griffith, 1967) also showed this constancy of the sex ratio. The mean of the ratios of the age-adjusted death rate of males and females in 12 cities was 1·64 and only in one city did the ratio differ significantly from this figure. Closer study revealed, however, that the value of the ratio in the combined material of all 12 cities differed by age over the 60-year age span investigated. Variations in the sex ratio with age had also been noted by Gordon, Crittenden and Haenszel (1961) in the United States. It was decided to analyse more extensive data to see whether the phenomenon was a general one.

### MATERIALS

A series of publications by Segi and his colleagues (Segi and Kurihara, 1962, 1964, 1966) give deaths by age, sex and site of cancer for 25 population groups in 24 countries for the 6 years 1958–63. The deaths by 5-year age groups from 25 through 84 years of males and of females from cancer of the stomach in each population during the 6 years were aggregated. The mean of the 1960 and 1961 populations in each age-sex group was used to calculate mean annual death rates. Rates based on deaths during 6 years were obtained for all countries except Chile, South Africa and Israel. For Chile, deaths during the 4 years 1960–63 were used with the 1961 census population as denominator. Deaths for 1963 were not available for South Africa and the rates are based on deaths during 5 years. The rates for Israel at ages 75–84 years refer to 1962–63 only but the rates at earlier ages were calculated in the same way as those of the other countries. The total number of deaths available for analysis was very large (455,626 males and

379,539 females) although in the younger age-groups the numbers by sex in several countries were too small to give stable rates even when the data for several years are pooled.

RESULTS

The mean annual death rates from gastric cancer by age and sex for each country are given in Table I and the ratio of male to female death rates by ages appear in Table II. The countries are arranged in rank order of age-adjusted death rates for males in 1962–63 from the highest (Japan) to the lowest (U.S. white). The means of the 25 sex-ratios by age group are also given in Table II.

It is at once apparent that the sex-ratio of mortality from gastric cancer varies with age. In young persons (25–29 years) the mean of the ratios is approximately one, rising steadily to a maximum at ages 55–59 years (2·23) and then falling progressively to 1·46 in the oldest age group. This trend is shown in all countries. Thus, for 19 of the 25 ratios the maximum value attained is found between 50 and 59 years and in no country does the maximum ratio appear before the age of 45 years or after the age of 70 years.

Japan, the Federal Republic of Germany, England and Wales, and United States (white) have sufficient numbers of deaths to yield relatively stable rates even in the youngest age group. They rank first, fifth, seventeenth and twenty-fifth respectively on the age-adjusted mortality of males. The curve of the sex-ratio by age is remarkably similar in the 4 countries despite the wide divergence in mortality (Fig. 1).

The relationship between the sex-ratio and age is thus independent of the level of mortality. The relationship also holds in countries where the secular trend of mortality has been sharply downwards (as in the United States) or declining more gradually (as in Germany or England and Wales) or has remained steady in recent years (as in Japan).

The rates analysed are effectively cross-sectional but the same trends can be seen in cohort data for England and Wales which have been published by Stocks (1958). The sex-ratios in cohort data for Scotland can be examined in tables prepared by Harley and Hytten (1966). These rates show the same pattern with ratios at younger ages and at older ages being lower than in middle life. For the most recent Scottish cohorts it is not possible to say whether the ratios have yet reached their maximum values but for the 1896 cohort the maximum value of the ratio(2·32) was for the age group 50–54 years. The 2 preceeding cohorts had lower maximum ratios (1·97 for the 1886 cohort and 1·63 for the 1876 cohort) appearing a decade earlier than the maximum of the 1896 cohort.

National mortality data do not distinguish between cancer arising in different parts of the stomach. Flamant *et al.* (1964) found that the sex-ratio after adjustment for age was higher for cancers of the fundus and cardia than for those of the pyloric region. In due course it should be possible to examine this aspect in other countries since the 1965 Revision of International Classification of Diseases (World Health Organization, 1967, as yet unpublished) allows this distinction to be made.

That the variation of sex-ratio in mortality can be explained in terms of differential survival is improbable. Pedersen (1964) has analysed survival of patients with gastric cancer in extensive material from a number of countries. He found only slight differences between the sexes in survival rates whether at younger

TABLE I.—*Mean Annual Death Rates from Gastric Cancer per* 100,000 *Population in* 5-*year Age Groups by Sex in* 24 *Countries*, 1958–63

| Country | Age group in years | | | | | | | | | | | |
|---|---|---|---|---|---|---|---|---|---|---|---|---|
| | 25–29 | 30–34 | 35–39 | 40–44 | 45–49 | 50–54 | 55–59 | 60–64 | 65–69 | 70–74 | 75–79 | 80–84 |
| | | | | | | Males | | | | | | |
| Japan | 2·9 | 7·5 | 16·2 | 33·4 | 65·0 | 117·3 | 205·8 | 316·7 | 460·2 | 568·1 | 60·15 | 499·7 |
| Chile | 3·3 | 7·7 | 12·1 | 29·7 | 62·5 | 110·6 | 169·9 | 281·7 | 441·3 | 633·7 | 790·5 | 668·5 |
| Finland | 1·2* | 3·4 | 7·4 | 13·7 | 29·2 | 55·2 | 117·1 | 198·2 | 314·4 | 432·7 | 523·4 | 588·6 |
| Austria | 0·8* | 2·5 | 5·4 | 16·2 | 28·6 | 53·2 | 102·7 | 169·0 | 279·0 | 417·5 | 564·9 | — |
| Germany, Federal Republic | 0·9 | 2·3 | 5·2 | 11·7 | 23·0 | 44·9 | 84·9 | 153·6 | 251·8 | 368·9 | 522·7 | 625·3 |
| Italy | 0·9 | 2·2 | 4·9 | 13·3 | 25·0 | 51·6 | 91·1 | 147·3 | 221·6 | 298·7 | 377·0 | 385·0 |
| Portugal | 1·2* | 3·9 | 8·2 | 17·4 | 31·5 | 57·0 | 98·8 | 139·8 | 204·9 | 237·1 | 276·1 | 212·8 |
| South Africa | — | 1·5* | 4·7* | 10·6 | 15·3 | 37·7 | 56·1 | 112·3 | 195·6 | 280·2 | 365·0 | 494·0 |
| Netherlands | 0·7* | 1·5 | 4·1 | 9·2 | 18·5 | 35·1 | 63·4 | 104·1 | 176·4 | 275·5 | 388·2 | 566·1 |
| Belgium | 0·7* | 1·7 | 4·4 | 10·2 | 19·1 | 37·1 | 66·9 | 113·6 | 178·1 | 257·0 | 384·3 | 519·2 |
| Switzerland | 0·7* | 1·5* | 3·1 | 6·8 | 16·5 | 30·4 | 57·9 | 112·2 | 187·5 | 282·3 | 424·6 | 525·5 |
| Norway | 0·5* | 1·4* | 3·4 | 10·6 | 16·9 | 36·7 | 64·9 | 120·0 | 174·1 | 272·0 | 339·4 | 486·3 |
| Scotland | 0·9* | 1·5* | 5·3 | 11·3 | 21·9 | 47·8 | 71·4 | 106·1 | 162·2 | 234·3 | 301·6 | 327·2 |
| Ireland | 1·4* | 1·4* | 4·3* | 12·0 | 23·4 | 41·5 | 67·9 | 101·8 | 155·1 | 201·9 | 250·0 | 293·9 |
| Northern Ireland | 2·8* | 3·3* | 5·4* | 7·5* | 24·4 | 49·6 | 75·7 | 109·4 | 166·7 | 214·1 | 273·8 | 309·2 |
| Sweden | 0·4* | 1·3* | 2·6 | 7·3 | 15·3 | 25·1 | 52·6 | 91·3 | 150·8 | 241·3 | 354·0 | 466·7 |
| England and Wales | 0·6 | 1·5 | 4·0 | 9·2 | 18·2 | 37·4 | 68·8 | 107·2 | 158·0 | 221·2 | 270·7 | 318·5 |
| Denmark | 0·7* | 0·9* | 3·7 | 7·2 | 12·9 | 26·0 | 53·1 | 89·9 | 148·8 | 224·1 | 332·2 | 465·2 |
| France | 0·3 | 0·9 | 2·6 | 6·0 | 14·5 | 30·0 | 53·7 | 98·0 | 159·0 | 231·5 | 327·5 | 385·7 |
| Israel | 0·7* | 1·4* | 2·1* | 6·1* | 12·6 | 20·8 | 54·2 | 79·2 | 150·2 | 201·1 | 316·4 | 379·8 |
| Canada | 0·5* | 1·4 | 3·1 | 6·0 | 12·5 | 24·2 | 45·1 | 71·7 | 116·8 | 165·6 | 237·4 | 313·0 |
| United States (non white) | 0·8 | 1·9 | 4·2 | 9·3 | 18·9 | 34·5 | 53·7 | 92·3 | 123·9 | 147·6 | 152·9 | 172·1 |
| New Zealand | 0·5* | 0·9* | 3·5* | 3·9* | 9·9 | 23·1 | 38·8 | 63·5 | 107·2 | 178·9 | 266·3 | 289·3 |
| Australia | 0·2* | 1·1 | 2·6 | 5·4 | 11·4 | 21·6 | 41·0 | 67·0 | 113·8 | 164·7 | 243·5 | 279·1 |
| United States (white) | 0·2 | 0·8 | 1·8 | 3·5 | 7·2 | 13·8 | 23·3 | 40·4 | 67·0 | 98·7 | 137·8 | 179·6 |
| | | | | | | Females | | | | | | |
| Japan | 4·1 | 10·2 | 17·7 | 30·0 | 43·7 | 65·8 | 95·7 | 142·8 | 211·0 | 279·0 | 316·8 | 293·1 |
| Chile | 2·7 | 6·4 | 10·4 | 18·5 | 35·8 | 56·8 | 95·7 | 161·4 | 265·1 | 367·3 | 542·9 | 548·6 |
| Finland | 1·2* | 2·3* | 7·2 | 10·2 | 18·4 | 23·9 | 47·1 | 88·6 | 158·5 | 243·3 | 342·2 | 404·2 |
| Austria | 0·6* | 2·4 | 3·7 | 9·3 | 14·5 | 23·6 | 43·7 | 79·2 | 131·2 | 236·0 | 364·2 | — |
| Germany, Federal Republic | 0·8 | 1·9 | 4·1 | 7·8 | 12·8 | 21·7 | 39·2 | 71·9 | 130·9 | 221·8 | 355·4 | 465·3 |
| Italy | 0·7 | 1·7 | 3·6 | 7·0 | 11·4 | 21·1 | 37·9 | 66·6 | 112·5 | 170·6 | 239·6 | 281·0 |
| Portugal | 1·1* | 2·4 | 4·3 | 9·8 | 15·6 | 30·0 | 45·8 | 73·4 | 112·2 | 145·4 | 173·8 | 169·2 |
| South Africa | 0·6* | 1·9* | 3·5* | 5·9 | 8·7 | 15·8 | 27·3 | 44·1 | 67·5 | 126·9 | 196·5 | 350·4 |
| Netherlands | 0·6* | 1·8 | 2·9 | 5·0 | 8·3 | 14·4 | 27·0 | 47·4 | 84·8 | 144·0 | 236·4 | 360·2 |
| Belgium | 0·9* | 1·5 | 2·7 | 6·5 | 9·6 | 17·4 | 32·6 | 55·5 | 91·3 | 160·5 | 255·8 | 390·8 |
| Switzerland | 0·6* | 1·2* | 2·0* | 4·5 | 7·6 | 14·8 | 25·7 | 52·2 | 96·8 | 166·6 | 267·8 | 404·7 |
| Norway | 0·5* | 1·2* | 5·5 | 7·9 | 12·3 | 17·4 | 33·0 | 54·8 | 87·8 | 130·1 | 224·3 | 313·2 |
| Scotland | 0·8* | 2·0* | 4·1 | 5·1 | 10·5 | 19·4 | 29·8 | 57·7 | 86·0 | 142·8 | 208·5 | 260·7 |
| Ireland | 0·9* | 0·6* | 2·7* | 7·2 | 11·1 | 21·7 | 40·4 | 62·9 | 99·7 | 145·3 | 217·2 | 239·2 |
| Northern Ireland | 1·1* | 0·7* | 3·5* | 5·1* | 9·2 | 19·4 | 34·4 | 53·6 | 91·2 | 155·6 | 214·7 | 233·0 |
| Sweden | 1·1* | 2·0 | 3·1 | 5·5 | 9·3 | 13·8 | 25·3 | 44·5 | 74·8 | 121·7 | 186·6 | 308·1 |
| England and Wales | 0·6 | 1·5 | 2·5 | 5·1 | 8·3 | 15·8 | 25·9 | 43·9 | 73·6 | 114·2 | 166·7 | 225·6 |
| Denmark | 0·1* | 1·4* | 2·8 | 5·0 | 9·4 | 11·4 | 21·9 | 42·0 | 71·3 | 131·2 | 237·7 | 366·1 |
| France | 0·4 | 0·7 | 1·6 | 3·5 | 6·4 | 12·3 | 22·1 | 39·3 | 69·7 | 118·2 | 185·5 | 254·4 |
| Israel | 0·3* | 1·3* | 3·0* | 6·2* | 11·4 | 14·1 | 28·4 | 40·1 | 95·2 | 123·9 | 220·0 | 281·3 |
| Canada | 0·5* | 0·9 | 2·6 | 3·7 | 6·7 | 9·6 | 18·5 | 30·2 | 50·3 | 78·5 | 124·1 | 174·6 |
| United States (non White) | 0·7 | 1·6 | 2·7 | 5·5 | 7·6 | 16·0 | 26·2 | 39·9 | 46·9 | 60·4 | 75·1 | 93·6 |
| New Zealand | — | 0·5* | 2·5* | 2·9* | 4·2* | 8·1 | 15·8 | 28·9 | 51·6 | 81·5 | 151·1 | 165·3 |
| Australia | 0·5* | 0·8* | 1·6 | 4·6 | 4·9 | 7·7 | 16·5 | 27·1 | 48·7 | 83·2 | 133·3 | 189·2 |
| United States (white) | 0·2 | 0·7 | 1·3 | 2·6 | 4·2 | 6·7 | 10·5 | 18·5 | 30·1 | 47·6 | 70·0 | 105·2 |

Rates indicated by an asterisk are based on fewer than 25 deaths.

TABLE II.—*Ratio of Male to Female Age-specific Death Rates at Ages* 25–84 *Years per* 100,000 *Population from Gastric Cancer in* 24 *Countries,* 1958–63

| | Age group in years | | | | | | | | | | | |
|---|---|---|---|---|---|---|---|---|---|---|---|---|
| | 25–29 | 30–34 | 35–39 | 40–44 | 45–49 | 50–54 | 55–59 | 60–64 | 65–69 | 70–74 | 75–79 | 80–84 |
| Mean of ratios | 1·04 | 1·16 | 1·33 | 1·59 | 1·93 | 2·22 | 2·23 | 2·14 | 2·06 | 1·85 | 1·63 | 1·46 |
| Japan | 0·8 | 0·7 | 0·9 | 1·1 | 1·5 | 1·8 | 2·2 | 2·2 | 2·2 | 2·0 | 1·9 | 1·7 |
| Chile | 1·2 | 1·2 | 1·2 | 1·6 | 1·8 | 2·0 | 1·8 | 1·7 | 1·7 | 1·7 | 1·5 | 1·2 |
| Finland | 1·0* | 1·5* | 1·0 | 1·3 | 1·6 | 2·3 | 2·5 | 2·2 | 2·0 | 1·8 | 1·5 | 1·5 |
| Austria | 1·3* | 1·0 | 1·5 | 1·7 | 2·0 | 2·2 | 2·4 | 2·1 | 2·1 | 1·8 | 1·6 | — |
| Germany, Federal Republic | 1·1 | 1·2 | 1·3 | 1·5 | 1·8 | 2·1 | 2·2 | 2·1 | 1·9 | 1·7 | 1·5 | 1·3 |
| Italy | 1·3 | 1·3 | 1·4 | 1·9 | 2·2 | 2·4 | 2·4 | 2·2 | 2·0 | 1·8 | 1·6 | 1·4 |
| Portugal | 1·1* | 1·6 | 1·9 | 1·8 | 2·0 | 1·9 | 2·2 | 1·9 | 1·8 | 1·6 | 1·6 | 1·3 |
| South Africa | 0·0* | 0·8* | 1·3* | 1·8 | 1·8 | 2·4 | 2·0 | 2·6 | 2·9 | 2·2 | 1·9 | 1·4 |
| Netherlands | 1·2* | 0·8 | 1·4 | 1·8 | 2·2 | 2·4 | 2·4 | 2·2 | 2·1 | 1·9 | 1·6 | 1·6 |
| Belgium | 0·8 | 1·1 | 1·6 | 1·6 | 2·0 | 2·1 | 2·0 | 2·0 | 2·0 | 1·6 | 1·5 | 1·3 |
| Switzerland | 1·2* | 1·2* | 1·6* | 1·5 | 2·2 | 2·0 | 2·2 | 2·2 | 1·9 | 1·7 | 1·6 | 1·3 |
| Norway | 1·0* | 1·2* | 0·6 | 1·3 | 1·4 | 2·1 | 2·0 | 2·2 | 2·0 | 2·1 | 1·5 | 1·6 |
| Scotland | 1·1* | 0·8* | 1·3 | 2·2 | 2·1 | 2·5 | 2·4 | 1·8 | 1·9 | 1·6 | 1·4 | 1·3 |
| Ireland | 1·6* | 2·2* | 1·6* | 1·7 | 2·1 | 1·9 | 1·7 | 1·6 | 1·6 | 1·4 | 1·2 | 1·2 |
| Northern Ireland | 2·6* | 4·7* | 1·5* | 1·5* | 2·6 | 2·6 | 2·2 | 2·0 | 1·8 | 1·4 | 1·3 | 1·3 |
| Sweden | 0·4* | 0·6* | 0·8 | 1·3 | 1·6 | 1·8 | 2·1 | 2·0 | 2·0 | 2·0 | 1·9 | 1·5 |
| England and Wales | 1·0 | 1·0 | 1·6 | 1·8 | 2·2 | 2·4 | 2·7 | 2·4 | 2·2 | 1·9 | 1·6 | 1·4 |
| Denmark | 7·0* | 0·6* | 1·3 | 1·4 | 1·4 | 2·3 | 2·4 | 2·1 | 2·1 | 1·7 | 1·4 | 1·3 |
| France | 0·8 | 1·3 | 1·6 | 1·7 | 2·3 | 2·4 | 2·4 | 2·5 | 2·3 | 2·0 | 1·8 | 1·5 |
| Israel | 2·3* | 1·1* | 0·7* | 1·0* | 1·1 | 1·5 | 1·9 | 2·0 | 1·6 | 1·6 | 1·4 | 1·4 |
| Canada | 1·0* | 1·6 | 1·2 | 1·6 | 1·9 | 2·5 | 2·4 | 2·4 | 2·3 | 2·1 | 1·9 | 1·8 |
| United States (non White) | 1·1 | 1·2 | 1·6 | 1·7 | 2·5 | 2·2 | 2·0 | 2·3 | 2·6 | 2·4 | 2·0 | 1·8 |
| New Zealand | — | 1·8* | 1·4* | 1·3* | 2·4* | 2·8 | 2·5 | 2·2 | 2·1 | 2·2 | 1·8 | 1·8 |
| Australia | 0·4* | 1·4* | 1·6 | 1·2 | 2·3 | 2·8 | 2·5 | 2·5 | 2·3 | 2·0 | 1·8 | 1·5 |
| United States (white) | 1·0 | 1·1 | 1·4 | 1·4 | 1·7 | 2·1 | 2·2 | 2·2 | 2·2 | 2·1 | 2·0 | 1·7 |

* Ratios based on fewer than 25 deaths for either sex have been omitted in calculating mean of ratios.

or at older ages. This conclusion is borne out by an analysis of incidence rates based on material from some of the larger cancer registries included in the recent publication by Doll, Payne and Waterhouse (1966). Thus, the curves of the sex ratios of the age-specific incidence rates for cancer of the stomach in Denmark, England and Wales (4 hospital regions), Finland, New York State (excluding New York City), Norway and Sweden all show pronounced maxima between the ages of 50–64 years with markedly lower ratios at younger and at older ages. A similar curve can be seen in incidence data from Puerto Rico (Martinez, 1967).

As is well known, certification of the cause tends to become less accurate in deaths at advanced ages and more so for female deaths than for male deaths. It seems unlikely however, that the pattern of sex-ratio by age can be explained on this basis. Not only is the same pattern shown by incidence data from cancer registries, when the diagnosis can be accepted as well-established, but it is also seen in the mortality data of countries with widely differing levels of medical services.

### DISCUSSION

There is thus a distinct pattern of variation with age in the sex-ratio of mortality from gastric cancer, which is seen in many different populations, is independent of the actual level of mortality and not apparently a cohort phenomenon. A similar pattern is seen in morbidity data from several large cancer registries.

FIG. 1.—Ratios of male to female death rates from gastric cancer at ages 25–84 years in four countries, 1958–63.

| | |
|---|---|
| ..................... Japan | ———————— England and Wales |
| — — — — — — Germany | —·—·—·— United States (white) |

FIG. 2.—Ratios of male to female death rates from cancer of large intestine and rectum at ages 25–84 years in four countries, 1958–63.

| | |
|---|---|
| ..................... Japan | ———————— England and Wales |
| — — — — — — Germany | —·—·—·— United States (white). |

It seems unlikely that some kind of general susceptibility to cancer is responsible for this pattern because cancer of other sites show a different pattern. Data for the same countries as those shown as in Fig. 1 were used to derive the sex-ratios of age specific death rates from cancer of the large intestine and rectum (Fig. 2). The variation with age is again similar in the 4 countries but quite unlike that shown by gastric cancer.

The data from Segi and Kurihara for cancer of the oesophegus have also been examined. The detailed results are not included here but these differ from those for gastric cancer and for cancer of the large intestine and rectum in 2 ways: first there is much greater variability between countries in the sex-ratio at any given age, and second, no common pattern of variation in the ratio with age can be seen in the several countries.

The ubiquity of the pattern in gastric cancer suggests that the curves are reproducing the behaviour of some relevant parameter of a general nature. In speculating what this might be, allowance must be made for an interval to elapse between the initiation of the cancer process and the fatal termination of the disease. For certain industrial cancers the latent interval is reasonably well established, the peak incidence occurring 15–20 years after exposure (Case *et al.*, 1954; Melik and Naryka, 1960). From a comparison of cohort mortalities from gastric cancer in England and Wales with the values predicted by a theoretical model, Stocks (1953) suggested an average interval of 18 years intervenes between the inception of the neoplastic process and death.

In relation to gastric cancer we may ask therefore what parameter in human biology has a sex-ratio of unity up to, say, age 10–15 years, rises to a maximum around the age of 40 years and declines thereafter to approach unity again about 70 years? Probably many nutritional and metabolic characteristics would satisfy these conditions. For example, Leitner, Moore and Sharman, (1960) give data for the levels of vitamin A in serum which show that up to adolescence the levels are similar in males and females, at ages 30–39 years the average level in males is about twice that in females but at older ages the levels by sex are again approximately equal.

Another example would be the total body content of potassium, a possible index of the total active cell-mass. Allen, Anderson and Langham, (1960) have reported estimates by age and sex which indicate a sex ratio of unity up to the age of puberty, an increase to a maximum in young adult life and a gradual decline thereafter to reach unity again in elderly persons.

However, a more suggestive parallel as regards gastric cancer, is provided by total food intake. Extensive data are available on calorie intake by age and sex of children and adolescents (for example, Burke *et al.*, 1959; Heald, Duegela and Brunschuyler, 1963). The ratio of male/female intake is about 1 up to the age of puberty at which time the ratio starts to increase so that by late adolescence males are consuming 50–65 per cent more than females. Information on food consumption on food consumption among older people suggests that at ages 55–60 years the average calorie intake of males is about 45–50 per cent above that of females but the differential tends to become progressively smaller with advancing age (Gillum and Morgan, 1955; Morgan, 1959; Steinkamp, Cohen and Walsh, 1965). The variability of calorie requirements of men in different occupations and of women during pregnancy and lactation make it difficult to estimate average values for whole populations in the prime working years. Harries, Hobson and

Hollingsworth (1960) have assembled data from numerous sources which show that the average daily calorie intake of active males may range from 2850 for bank officials, to 4030 for miners, to 5026 for army troops during exercises. An average value given for housewives was 2100 and from 2354 to 2633 for pregnant women. It would thus appear that at the peak of his active working life the average male is consuming substantially more calories than the average female of the same age.

If exposure to a food-borne carcinogen is a necessary condition (though not perhaps a sufficient condition) for malignant change to occur, the probability of developing gastric cancer will depend first, on the quantity of food ingested and second, on the concentration of carcinogen in food. The variation in the sex-ratio of mortality, it is suggested, reflects variations in the former while the concentrations of the carcinogen would determine the level of mortality in a population. Certain implications of this hypothesis may be tentatively examined in relation to what is known about the epidemiology of gastric cancer.

Many studies have been conducted comparing the diets of patients with gastric cancer and those of controls variously selected (Stocks and Karn, 1933; Wynder *et al.*, 1963; Acheson and Doll, 1964; Higginson, 1966). These have not given any strong and consistent indications to incriminate any one item of diet. Also, no one common food product can be said to predominate in the average diet of all countries with a high mortality. These facts might be explained if a number of separate foodstuffs could serve as vechicles for the postulated carcinogen.

The well-known socio-economic gradient in gastric cancer suggests that the carcinogen is more likely to be found in cheap rather than expensive foods. The SMRs of males in Class I and Class V in England and Wales in 1951 were 57 and 130 respectively, a difference of 2·3 times (Registrar General, 1957). In the United States the disparity was even larger, the SMRs at ages 20–64 years being 56 and 157 for professional workers and labourers respectively (Guralnick, 1963). Total food consumption of males by occupation may have differed but hardly to this extent. On the other hand the difference between social classes in average consumption of certain items of food would probably have been greater than the differences in total consumption. Wynder *et al.*, (1963) point to the association between gastric cancer and diets high in carbohydrate content and a recent international comparison by Hakama and Saxén (1967) confirms the relationship. Haenszel (1958) has discussed at length the decline in mortality in the United States in relation to changes in food habits, and in particular the way in which citrus fruits have displaced apples, and lettuce has displaced cabbage in the national dietary. It may however, be noted that *per capita* consumption of carbohydrates in the United States has also been declining progressively since the turn of the century. The trend has been most marked for complex carbohydrate foods; for example, the quantity of potatoes available to retail markets was halved, from 217·8 lb to 110 lb *per capita*, between 1899 to 1961 (Antar, Ohlson and Hodges, 1964). By contrast, in the United Kingdom, where the decline in gastric cancer has been much less marked than in the United States, according to Hollingsworth and Greaves (1967) potato consumption decreased relatively little between 1910 and 1962. It may be significant that of the many separate foodstuffs listed by Haenszel (1958) the one showing the largest number of positive relationships with the epidemiological criteria used by him should have been potatoes.

A coherent hypothesis of aetiology must be able to accommodate the curious relationship between the occurrence of gastric cancer and certain characteristics of soil, noted in the Netherlands (Tromp and Diehl, 1955) and in Japan (Kurokawa, 1961) as well as in Britain where detailed studies have been made by Stocks and Davies (1960, 1964). The highly localized nature of the associations found by them might be explained by postulating that certain types of soil favour the production of a carcinogen for which a local-grown foodstuff serves as the vechicle. The negative findings reported from Iceland by Armstrong (1964) while tending to exclude other possible explanations for the association with soil do not conflict with this suggestion. In some areas suspicion might well attach to potatoes (Griffith, 1963). In regions where potatoes do not figure prominently in the diet the hypothesis would require that other foodstuffs are liable to contamination by the postulated carcinogen.

The observation by Butler and Barnes (1966) that adenocarcinoma of the glandular stomach can occur in rats fed a ration containing contaminated groundnuts is thus of considerable interest. Many agricultural products (barley, beans, coffee, corn, peanuts, potatoes, rice, sorghum, wheat) are liable to fungal contamination with the production of toxic metabolites some of which may be extremely potent carcinogens (Wogan, 1965; Barnes and Butler, 1964). There is as yet no evidence that these facts have any bearing on human cancer. Nevertheless several features of its epidemiology might be explained on the hypothesis that gastric cancer in man is caused by a carcinogenic metabolite of an ubiquitous soil fungus produced on any one of a variety of suitable carbohydrate substrates.

Since the disease is rare in those parts of the world where food is most liable to contamination with *Aspergillus flavus* it seems likely that the postulated mycotoxin would be derived from some other fungus. Both the species and the abundance of fungal growth can vary with the character of the soil (Joffe and Borut, 1966). In the search for likely species useful leads might be forthcoming from a consideration of the soil types associated with gastric cancer (Davies and Griffith, 1954) and their mineral content (Stocks and Davies, 1960, 1964) in relation to the growth requirements of different fungi.

### SUMMARY

1. The sex ratio of mortality from gastric cancer shows a remarkably similar pattern in all countries for which recent data are available.

From a value close to one at younger ages the ratio rises to a maximum of two or more around the age of 60 years declining thereafter to approach unity again at advanced ages.

2. This pattern appears to be peculiar to gastric cancer since it is not seen in cancer of other parts of the intestinal tract. It is independent of the level of mortality and it is unlikely to be due to age-sex differentials in diagnostic accuracy or case fatality as data from several large cancer registries show similar trends.

3. The ubiquity of the pattern suggests the curve is reproducing the behaviour of some relevant parameter of a general nature.

Although more complete information would be desirable, the available data suggests that the sex ratio of total calorie consumption would fit the observed pattern after allowing for an appropriate latent interval.

4. Some speculations relative to aetiology are offered based on the hypothesis that a food-borne carcinogen is a necessary condition for the disease to occur.

### REFERENCES

ACHESON, E. D. AND DOLL, R.—(1964) *Gut*, **5**, 128.
ALLEN, T. H., ANDERSON, E. C. AND LANGHAM, W. H.—(1960) *J. Geront.*, **15**, 348.
ANTAR, M. A., OHLSON, M. A. AND HODGES, R. E.—(1964) *Am. J. clin. Nutr.*, **14**, 169.
ARMSTRONG, R. W.—(1964) *Acta Agric. scand.*, **14**, 65.
BARNES, J. M. AND BUTLER, W. H.—(1964) *Nature, Lond.*, **202**, 1016.
BURKE, B. S., REED, R. B., VAN DEN BERG, A. S. AND STUART, H. C.—(1959) *Pediatrics, N.Y.*, **24**, 922.
BUTLER, W. H. AND BARNES, J. M.—(1966) *Nature Lond.*, **209**, 90.
CASE, R. A. M., HOSKER, M. E., McDONALD, D. B. AND PEARSON, J. T.—(1954) *Br. J. ind. Med.*, **11**, 75.
DAVIES, R. I. AND GRIFFITH, G. W.—(1954) *Br. J. Cancer*, **8**, 56.
DOLL, R.—(1966) *Gastroenterology*, **86**, 320.
DOLL, R., PAYNE, P. AND WATERHOUSE, J.—(1966) ' Cancer incidence in five continents ' (Publication of the International Union against Cancer). Berlin (Springer-Verlag).
FLAMANT, R., LASSERRE, O., LAZAR, P., LEGUERINAIS, J., DENOIX, P. AND SCHWARTZ, D.—(1964) *J. natn. Cancer Inst.*, **32**, 1309.
GILLUM, H. L. AND MORGAN, A. F.—(1955) *J. Nutr.*, **55**, 265.
GORDON, T., CRITTENDEN, M. AND HAENSZEL, W.—(1961) *Natn. Cancer Inst. Monogr.*, No. 6, Part II.
GRIFFITH, G. W.—(1963) ' Stomach Cancer and soil ' in ' Cancer Progress ', edited by R. W. Raven. London (Butterworth).
GURALNICK, L.—(1963) ' Mortality by Occupation Level and Cause of Death among Men 20 to 64 Years of Age: United States, 1950 ' *Vital Statist. spec. Rep.*, **53**, No. 5. Washington, D.C.
HAENSZEL, W.—(1958) *J. natn. Cancer Inst.*, **21**, 213.
HAKAMA, M. AND SAXÉN, E. A.—(1967) *Int. J. Cancer*, **2**, 265.
HARLEY, J. L. AND HYTTEN, C. A.—(1966) ' Death Rates by Site, Age and Sex. 1911–1960, Scotland, ' Department of Public Health and Social Medicine, University of Aberdeen, and Chester Beatty Research Institute, London.
HARRIES, J. N., HOBSON, E. A. AND HOLLINGSWORTH, D. F.—(1960) *Proc. Nutr. Soc.*, **21**, 157.
HEALD, F. P., DAUGELA, M. AND BRUNSCHUYLER, P.—(1963) *New Engl. J. Med.*, **268**, 243.
HIGGINSON, J.—(1966) *J. natn. Cancer Inst.*, **37**, 527.
HOLLINGSWORTH, D. F. AND GREAVES, J. P.—(1967) *Am. J. clin. Nutr.*, **20**, 65.
JOFFE, A. Z. AND BORUT, S. Y.—(1966) *Mycologia*, **58**, 629.
KUROKAWA, T.—(1961) *Acta Un. int. Cancr.*, **17**, 848.
LEITNER, Z. A., MOORE, T. AND SHARMAN, I. M.—(1960) *Br. J. Nutr.*, **14**, 157.
MARTINEZ, I. (editor)—(1967) ' Cancer in Puerto Rico, 1950–1964 ' San Juan (Central Cancer Registry, Department of Health).
MELIK, W. F. AND NARYKA, J. J.—(1960) *Acta Un. int. Cancr.*, **16**, 277.
MORGAN, A. F.—(1959) ' Nutritional Status USA ', *Calif. agric. exp. Stn. Bull.*, **769**.
PEDERSEN, E.—(1964) *Natn. Cancer Inst. Monogr.*, **15**, 241.
PUFFER, R. R. AND GRIFFITH, G. W.—(1967) ' Patterns of Urban Mortality ', Scientific Publication 151. Washington (Pan American Health Association).
REGISTRAR GENERAL—(1957) ' Decennial Supplement, England and Wales 1951. Occupational Mortality '. London (H.M. Stationery Office).
SEGI, M. AND KURIHARA, M.—(1962) ' Cancer Mortality for Selected Sites in 24 Countries ', No. 2 (1958–1959). Sendai, Japan (Department of Public Health, Tohoku University School of Medicine.)—(1964) ' Cancer Mortality for Selected Sites in 24 Countries ' No. 3 (1960–1961). Sendai, Japan (Department of Public

Health, Tohoku University School of Medicine).—(1966) 'Cancer Mortality for Selected Sites in 24 Countries' No. 4 (1962–1963). Sendai, Japan (Department of Public Health, Tohoku University School of Medicine.)

STEINKAMP, R. C., COHEN, N. L. AND WALSH, H. E.—(1965) *J. Am. diet. Ass.*, **46**, 103.

STOCKS, P.—(1953) *Br. J. Cancer*, **7**, 407.—(1958) 'Statistical Investigations concerning the Causation of Various Forms of Human Cancer' in 'Cancer', edited by R. W. Raven. London (Butterworth) Vol. 3.

STOCKS, P. AND DAVIES, R. I.—(1960) *Br. J. Cancer*, **14**, 8.—(1964) *Br. J. Cancer*, **18**, 14

STOCKS, P. AND KARN, M.—(1933) *Ann. Eugen.*, **5**, 237.

TROMP, S. W. AND DIEHL, J. C.—(1955) *Br. J. Cancer*, **9**, 349.

WOGAN, G. N.(editor)—(1965) 'Mycotoxins in Foodstuffs', Cambridge, Mass. (M.I.T. Press).

WYNDER, E. L., KMET, J., DUNGAL, N. AND SEGI, M.—(1963) *Cancer N.Y.*, **16**, 1461.